# Letter to the Editor: Dysphagia Screening in an Aging Population: A Scoping Review of Structural Gaps Between Triage and Comprehensive Evaluation in International and Japanese Practices

**DOI:** 10.1002/jgf2.70168

**Published:** 2026-09-16

**Authors:** Insbat Zafar

**Affiliations:** ^1^ Fatima Jinnah Medical University Lahore Pakistan

Dear Editor,

1

I commend Ueda and Nohara for their insightful scoping review, “Dysphagia Screening in an Aging Population: A Scoping Review of Structural Gaps Between Triage and Comprehensive Evaluation in International and Japanese Practices” [1]. This timely work addresses critical triage‐to‐evaluation gaps in geriatric care and implementing targeted rehabilitation therapies. However, it presents several methodological limitations that warrant discussion.

First, the study omits a formal methodological quality appraisal of the included literature. Although quality appraisal is optional in scoping reviews, omitting it makes it difficult to distinguish high‐integrity, evidence‐based triage protocols from poorly reported institutional studies and prevents clinicians from confidently translating these findings into diverse rehabilitation settings. As Estupiñán‐Artiles et al. [2] emphasize, incorporating a structured quality assessment is vital to validate the clinical reliability of synthesized screening frameworks.

Second, by excluding gray literature, the review misses localized regional guidelines and municipal health policies, thereby oversimplifying the complex organizational structures of national healthcare systems. In countries like Japan, elderly community‐level triage is frequently guided by unpublished institutional protocols rather than peer‐reviewed papers. Andrews and Pillay [3] highlight that clinical dysphagia service structures are heavily documented in gray literature, making its exclusion a major gap in mapping actual global practices.

Third, the authors fail to stratify the structural gaps by distinct clinical settings, such as acute stroke units versus long‐term care facilities. Grouping these highly diverse environments together obscures setting‐specific operational realities and clinical resource differences. Consequently, the unique clinical barriers of low‐resource environments are completely overlooked. Hazelwood et al. [4] demonstrate that dysphagia screening workflows and clinician competencies are highly setting‐dependent, indicating that a non‐stratified comparison masks critical institutional barriers.

Fourth, the review evaluates screening as a static, individual role rather than analyzing dynamic interdisciplinary co‐management, which is fundamental to preventing silent aspiration and ensuring patient safety. Clinical decisions regarding oral intake in older adults are rarely restricted to linear, single‐professional screening sequences. Boaden et al. [5] point out that successful dysphagia care pathways rely heavily on collaborative, multi‐professional team dynamics and shared clinical decision‐making rather than isolated bedside handoffs.

To overcome these limitations, future studies should incorporate formal quality appraisal tools to evaluate evidence strength. Search strategies must expand to include gray literature and national clinical guidelines to capture localized protocols across varied economic regions. Additionally, researchers must stratify swallowing workflows by specific clinical settings and utilize longitudinal, mixed‐methods designs to observe how interdisciplinary team dynamics and shared decision‐making influence patient outcomes over time.

In conclusion, while Ueda and Nohara [1] provide an admirable synthesis of dysphagia screening pathways, addressing these key limitations—lack of quality appraisal, exclusion of gray literature, absence of setting stratification, and neglect of team dynamics—is crucial to enhance the accuracy, clinical relevance, and global translational potential of future research and clinical guidelines in geriatric swallowing care.

## Author Contributions

**Insbat Zafar:** methodology, validation, software, resources, writing – review and editing.

## Funding

The author has nothing to report.

## Disclosure

Guarantor Statement: All authors have read and approved the final version of the manuscript. They took complete responsibility for the data's integrity and the data analysis's accuracy.

Transparency Statement: The Authors affirm that this manuscript is an honest, accurate, and transparent account of the study being reported, that no important aspects of the study have been omitted, and that any discrepancies from the study as planned (and if relevant, registered) have been explained.

## Conflicts of Interest

The author declares no conflicts of interest.

## Data Availability

Data sharing does not apply to this article as no datasets were generated during the current study; all data were sourced from published literature.

## References

[1] A. Ueda and K. Nohara , “Dysphagia Screening in an Aging Population: A Scoping Review of Structural Gaps Between Triage and Comprehensive Evaluation in International and Japanese Practices,” Current Study. 10.1002/jgf2.70150 PMC13365870 42453638

[2] C. Estupiñán‐Artiles , J. Regan , and C. Donnellan , “Dysphagia Screening in Residential Care Settings: A Scoping Review,” International Journal of Nursing Studies 114 (2021): 103813, 10.1016/j.ijnurstud.2020.103813. 33220569

[3] M. Andrews and M. Pillay , “Dysphagia Services in Resource‐Constrained Settings: A Systematic Review,” South African Journal of Communication Disorders 66, no. 1 (2019): a611, 10.4102/sajcd.v66i1.611.

[4] R. J. Hazelwood , G. F. Robinson , G. W. Wolford , and R. F. Smith , “Assessment of Dysphagia Management Competence Among Healthcare Providers: A Scoping Review,” Dysphagia 40, no. 6 (2025): 1381–1398, 10.1007/s00455-024-10700-1. 40437232 PMC12662856

[5] E. Boaden , J. Burnell , L. Hives , et al., “Screening for Dysphagia in Acute Stroke: A Systematic Review and Meta‐Analysis of Diagnostic Accuracy,” Journal of Clinical Nursing 30, no. 13–14 (2021): 1832–1846, 10.1111/jocn.15734.

